# Quantifying pruning impacts on olive tree architecture and annual canopy growth by using UAV-based 3D modelling

**DOI:** 10.1186/s13007-017-0205-3

**Published:** 2017-07-06

**Authors:** F. M. Jiménez-Brenes, F. López-Granados, A. I. de Castro, J. Torres-Sánchez, N. Serrano, J. M. Peña

**Affiliations:** 1 Institute for Sustainable Agriculture, CSIC, 14004 Córdoba, Spain; 2 Institute of Agricultural Research and Training (IFAPA), 14004 Córdoba, Spain; 3 Institute of Agricultural Sciences, CSIC, 28006 Madrid, Spain

**Keywords:** Crown volume, Remote sensing, Unmanned aerial vehicle, Object-based image analysis, Precision agriculture

## Abstract

**Background:**

Tree pruning is a costly practice with important implications for crop harvest and nutrition, pest and disease control, soil protection and irrigation strategies. Investigations on tree pruning usually involve tedious on-ground measurements of the primary tree crown dimensions, which also might generate inconsistent results due to the irregular geometry of the trees. As an alternative to intensive field-work, this study shows a innovative procedure based on combining unmanned aerial vehicle (UAV) technology and advanced object-based image analysis (OBIA) methodology for multi-temporal three-dimensional (3D) monitoring of hundreds of olive trees that were pruned with three different strategies (traditional, adapted and mechanical pruning). The UAV images were collected before pruning, after pruning and a year after pruning, and the impacts of each pruning treatment on the projected canopy area, tree height and crown volume of every tree were quantified and analyzed over time.

**Results:**

The full procedure described here automatically identified every olive tree on the orchard and computed their primary 3D dimensions on the three study dates with high accuracy in the most cases. Adapted pruning was generally the most aggressive treatment in terms of the area and volume (the trees decreased by 38.95 and 42.05% on average, respectively), followed by trees under traditional pruning (33.02 and 35.72% on average, respectively). Regarding the tree heights, mechanical pruning produced a greater decrease (12.15%), and these values were minimal for the other two treatments. The tree growth over one year was affected by the pruning severity and by the type of pruning treatment, i.e., the adapted-pruning trees experienced higher growth than the trees from the other two treatments when pruning intensity was low (<10%), similar to the traditionally pruned trees at moderate intensity (10–30%), and lower than the other trees when the pruning intensity was higher than 30% of the crown volume.

**Conclusions:**

Combining UAV-based images and an OBIA procedure allowed measuring tree dimensions and quantifying the impacts of three different pruning treatments on hundreds of trees with minimal field work. Tree foliage losses and annual canopy growth showed different trends as affected by the type and severity of the pruning treatments. Additionally, this technology offers valuable geo-spatial information for designing site-specific crop management strategies in the context of precision agriculture, with the consequent economic and environmental benefits.

**Graphical Abstract:**

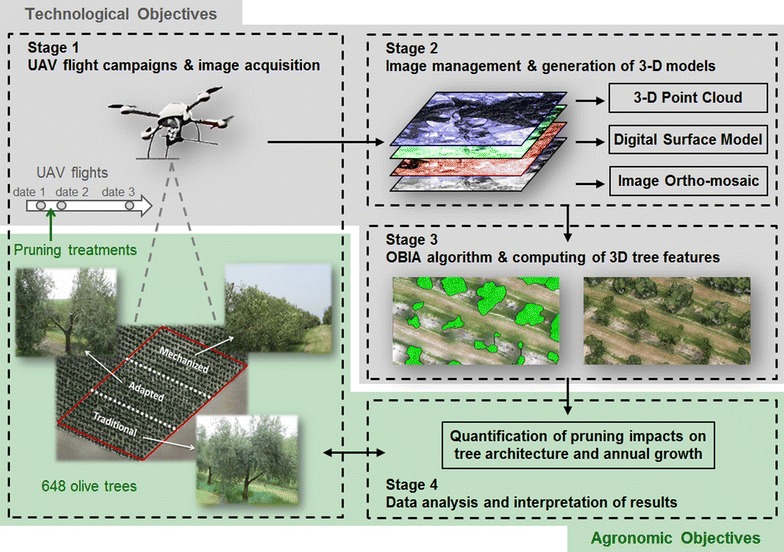

## Background

Crop viability essentially relies on the management strategy adopted by the farmer. Among the tasks that impact orchard production, tree pruning remains as a costly practice with important implications for harvest [1], nutrition, pest and disease control, and irrigation strategies [2]. The pruning type and its intensity modify the tree crown to differing degrees of severity, which notably affects the tree physiology and, consequently, the fruit quantity and quality [3, 4]. Investigations on tree pruning usually involve the characterization of the tree architecture by measuring several geometric features of the crown. The conventional method consists in using a ruler to measure the primary dimensions of the tree (e.g., the tree height and its primary axis) and, next, estimating the canopy area and the crown volume either by applying equations that treat the trees as regular polygons or by applying empirical models [5]. Obviously, this task is very tedious; it requires intensive fieldwork and usually generates inconsistent results due to the irregular geometry of the tree crown [6].

Current advances in sensors and geo-spatial technologies offer an alternative to hands-on measurement tasks. Rosell and Sanz [7] described the following techniques: ultrasound, digital photographic techniques, light sensors, high-resolution radar images, high-resolution X-ray computed tomography, stereovision and LiDAR sensors. However, although some of these techniques, primarily terrestrial LiDAR laser scanning and stereovision systems, are very precise at measuring crop architecture [8, 9], they still pose some limitations under real agricultural scenarios that are usually characterized by large spaces and rugged areas. In these cases, unmanned aerial vehicles (UAVs) or drones have become a cost-effective tool for collecting continuous crop information at the field scale. The advantages of the UAVs in comparison to the traditional remote-sensing platforms are attributed to their lower cost, greater flexibility in flight scheduling and their capacity to collect remote images with much higher spatial resolution [10–12]. In addition, because the UAVs can fly at low altitude and acquire images with high overlaps, these images can be processed with automatic photo-reconstruction software and be used to produce a Digital Surface Model (DSM) of the flight area, i.e., the three dimensions (3D) of the topography and all the elements (e.g., trees) over the surface [13]. As a consequence, recent investigations have focused on evaluating the quality of UAV-based 3D models of tree plantations, and they have reported satisfactory results for olive trees [14–16], palm trees [17] and *Pinus pinea* [18]. For example, by comparing UAV-based estimations of olive trees to on-ground measurements, Torres Sánchez et al. [15] obtained coefficients of determination (R^2^) of 0.94, 0.90 and 0.65 for projected canopy area, tree height and crown volume, respectively.

To seize on all the benefits of the UAV capacity for collecting detailed information over large areas at a spatial resolution of a few centimeters, it is essential to develop and apply robust and automatic image analysis tools that are capable of computing a huge amount of crop data to produce useful maps for crop monitoring or other agronomic objectives. The object-based image analysis (OBIA) paradigm includes a wide array of techniques that offer a high level of automation and adaptability, improving on some of the limitations of pixel-based methods [19]. OBIA is based on two primary stages, called segmentation and classification. In the first stage, adjacent pixels with homogenous digital values are grouped as “objects”, which are used as the basic elements of analysis and classification [20]. In the second stage, OBIA combines the spectral, topological and contextual features of these objects to successfully address complicated classification issues, e.g., in rangelands [21, 22], or urban areas [23].

**Fig. 1:**
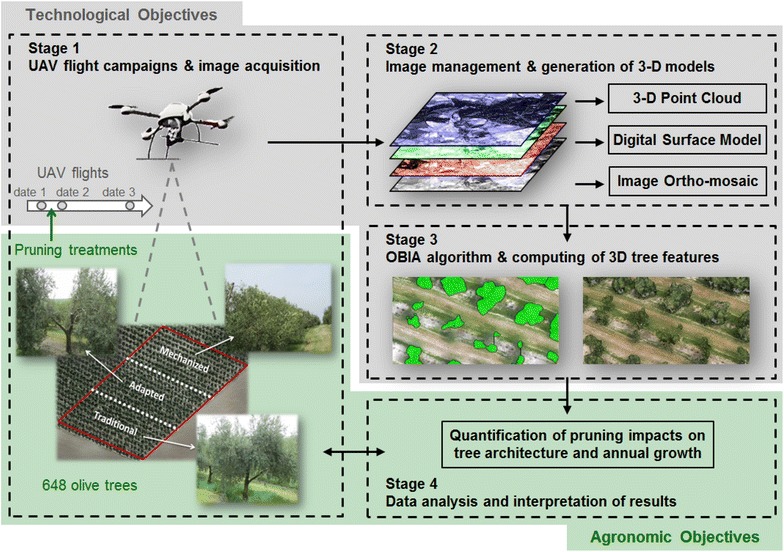
Graphical scheme of the stages and specific technological and agronomic objectives of this investigation

The combined use of UAV images, 3D models and OBIA procedures offers new opportunities for the high-throughput monitoring of crop conditions at the level of individual plants or trees [24]. Therefore, this study takes advantage of this geo-spatial technology to compute the 3D geometric features of hundreds of olive trees with the ultimate objective of quantifying the pruning impact on the tree architecture and tree growth. Three different pruning treatments were evaluated by comparing a multi-temporal UAV-based dataset that was collected before tree pruning, after tree pruning and one year after tree pruning. The specific objectives of this research were separated in two linked sections (Fig. 1) as follows: (a) technological objectives, which involved the description and evaluation of the full procedure to acquire remote images with the UAV and to generate the image-based 3D tree models. These objectives included the development and implementation of an innovative OBIA algorithm with the capacity to automatically classify and identify every tree of the olive grove and to compute their position, projected area, height, and volume; and (b) agronomic objectives, which aimed to explore and interpret the temporal variability that was measured within the olive grove as affected by every applied pruning treatment. Additionally, the potential uses of the valuable dataset and maps obtained with this technology were also discussed, including applications for physiological and agronomical studies as well for designing site-specific crop management strategies in the context of precision agriculture.

## Results

### Technological objectives: multi-temporal quantification of the tree 3D features (location, projected canopy area, tree height and crown volume) at the field scale

The OBIA algorithm that was developed for this investigation automatically identified all the olive trees and reported their geographic coordinates, projected areas, heights and volumes on the three study dates (Table 1). The algorithm also accounted for the relative position of the trees, i.e. their row number (from 1 to 27) and their order in the row (from 1 to 24), which facilitated their localization within the field.

**Table 1:** A sample of the output dataset delivered by the customized OBIA algorithm

Tree ID	Row	Column	Central coordinates^a^		Pruning treatment	Projected canopy area (m^2^)			Tree height (m)			Crown volume (m^3^)		
			X	Y		Date 1	Date 2	Date 3	Date 1	Date 2	Date 3	Date 1	Date 2	Date 3
1	1	1	485,726	4,217,762	Traditional	15.38	12.44	17.24	3.81	3.72	3.75	44.09	34.74	43.16
2	1	2	485,732	4,217,760	Traditional	15.88	9.61	16.69	4.10	4.01	4.14	47.75	27.52	44.68
3	1	3	485,738	4,217,757	Traditional	13.98	10.77	14.58	3.91	3.62	3.70	35.42	26.68	34.68
…	…	…	…	…	…	…	…	…	…	…	…	…	…	…
217	10	1	485,786	4,217,808	Adapted	15.34	5.16	11.13	4.37	4.04	4.43	43.81	13.28	31.43
218	10	2	485,789	4,217,806	Adapted	14.96	5.46	12.26	3.81	3.80	3.97	43.81	15.15	35.45
219	10	3	485,795	4,217,803	Adapted	12.64	7.62	13.02	4.53	3.77	3.45	31.73	18.99	27.43
…	…	…	…	…	…	…	…	…	…	…	…	…	…	…
433	19	1	485,840	4,217,852	Mechanical	7.00	5.75	7.20	3.85	3.48	3.83	18.78	15.67	17.68
434	19	2	485,846	4,217,851	Mechanical	10.11	10.00	13.64	4.31	3.58	3.79	27.28	26.73	31.97
435	19	3	485,850	4,217,848	Mechanical	13.55	13.46	14.93	4.17	3.54	3.61	43.87	39.38	42.49
…	…	…	…	…	…	…	…	…	…	…	…	…	…	…
648	27	24	486,017	4,217,841	Mechanical	6.06	5.93	6.30	3.48	3.34	3.54	15.27	14.52	15.46

^a^Coordinate system: UTM, zone 30 N, datum WGS84

The computed values were generally consistent on the three dates, decreasing from date 1 to date 2 as a result of the pruning operation, and increasing from date 2 to date 3 due to the tree development that occurred over one year. On average, the projected canopy areas varied from a range of 11.6–14.7 m^2^ on date 1 (before pruning) to 8.4–10.7 m^2^ on date 2 (after pruning) and to 13.7–15.1 m^2^ on date 3 (1-year after pruning). Similarly, the tree heights varied over a range from 3.9 to 4.1 m (on date 1) to 3.4–4.1 m (on date 2) and to 3.3–4.2 m (on date 3), and the crown volumes varied over a range from 31.9 to 42.7 m^3^ (on date 1) to 22.7–28.1 m^3^ (on date 2) and to of 32.8–41.0 m^3^ (on date 3).

However, detailed observations of the full dataset revealed incorrect dimensions for some trees, which could be attributed to errors that occurred during the DSM generation. Therefore, the quality of the DSM created on the three dates was evaluated by visually comparing every tree perimeter that was defined by the OBIA algorithm (which was based on the DSM information) and the real tree perimeter observed in the orthomosaicked image. The accuracy that was achieved for the full 3D tree photo-reconstruction procedure varied as affected by the type of pruning treatment and the flight date (Table 2).

**Table 2:** Number and percentage of trees correctly photo-reconstructed on one of the three study dates (columns Date 1, Date 2 and Date 3) and on all the three study dates (column 1–2–3)

Pruning treatment	Number of trees	Date 1	Date 2	Date 3	Dates 1–2–3
Mechanical	216	185 (85.7%)	208 (96.3%)	191 (88.4%)	177 (81.9%)
Adapted	216	200 (92.6%)	163 (75.5%)	177 (81.9%)	161 (74.5%)
Traditional	216	200 (92.6%)	180 (83.3%)	190 (88.0%)	174 (80.6%)

Flight dates: Date 1 (Before pruning); Date 2 (After pruning); Date 3 (1-year after pruning)

When the three dates were jointly considered, the most accurate olive tree photo-reconstruction was obtained for mechanical pruning (MP). However, for individual dates, the worst and best results for the three treatments were all obtained on date 2, after pruning. These values varied from 75.5% at the adapted pruning (AP) parcel, to 83.3% at the traditional pruning (TP) parcel and to 96.3% at the MP parcel. This finding could be due to the specific characteristics of each treatment. The MP removed protruding branches, which produced a tree shape that was more uniform than that of the other pruning treatments, thus facilitating the task of building the 3D point cloud field geometry and consequently, the correct definition and photo-reconstruction of the MP tree edges. In the AP treatment, pruning drastically cut back the crown biomass of the trees, which increased the overall tree shape heterogeneity. As a result, the accuracy of the photo-reconstruction of some of the trees decreased. These results indicate that the pruning treatment affected the tree architecture and crown size, and it might have also positively or negatively affected the quality of the UAV-based 3D geo-spatial products. Additionally, no correlation between every tree location and photo-reconstruction errors was found at the orchard scale, which also indicates that other factors with respect to the weather conditions (e.g., wind or clouds) or operational issues (e.g., flight altitude, orientation of sensor axes or UAV velocity) could apparently produce slight random changes at the moment of image shooting. As a result of this evaluation, 512 trees (approximately 80% of the total) that were correctly photo-reconstructed on the three dates were studied in the subsequent analysis, and the rest of the trees were discarded to avoid imprecise conclusions in the context of the agronomic objectives proposed in this investigation.

**Fig. 2:**
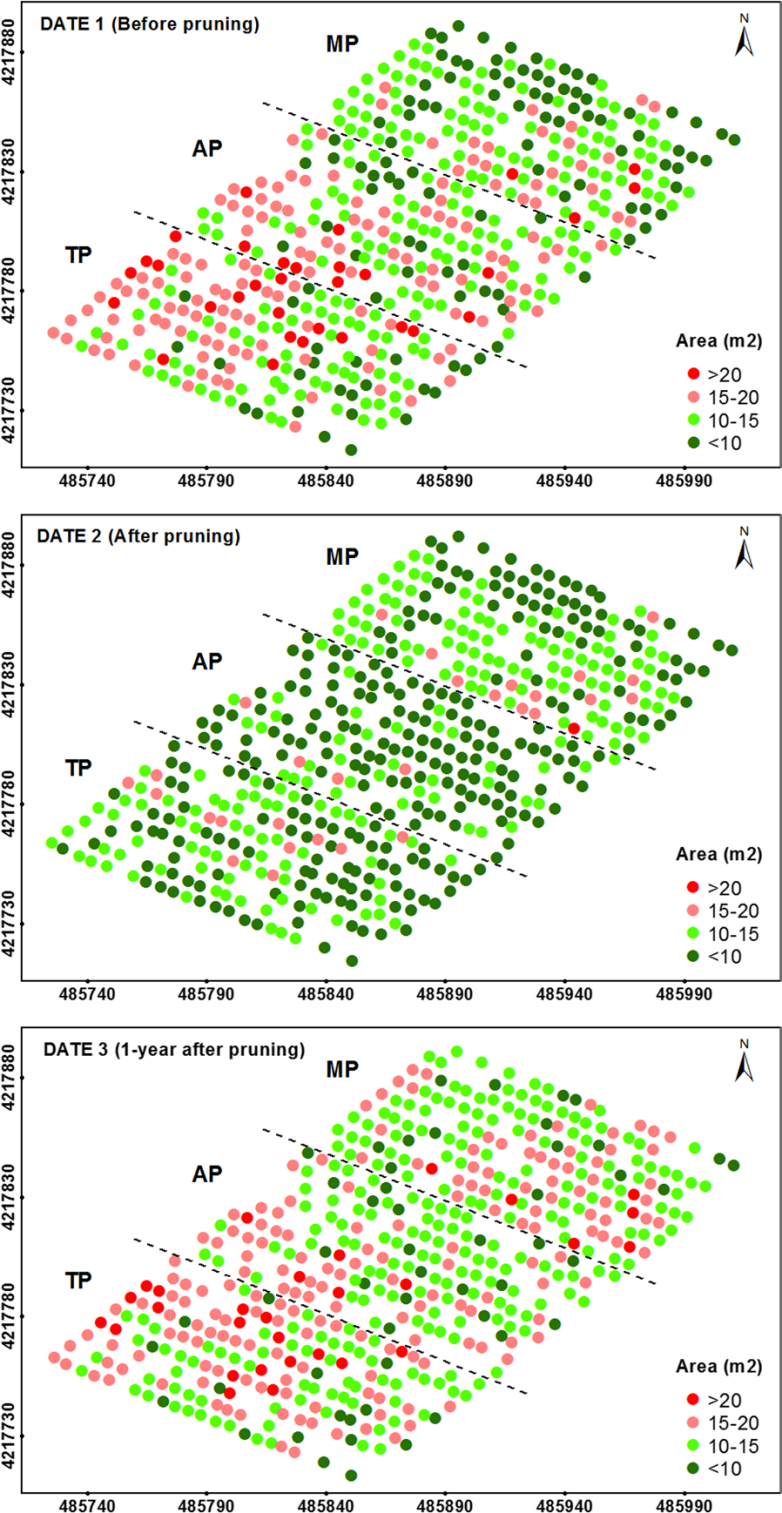
Four-level representation of the projected tree canopy areas as computed on the three study dates. The *letters* indicate the pruning treatments as follows: TP (traditional), AP (adapted), and MP (mechanical). In the axes, coordinate system UTM zone 30 N, datum WGS84

**Fig. 3:**
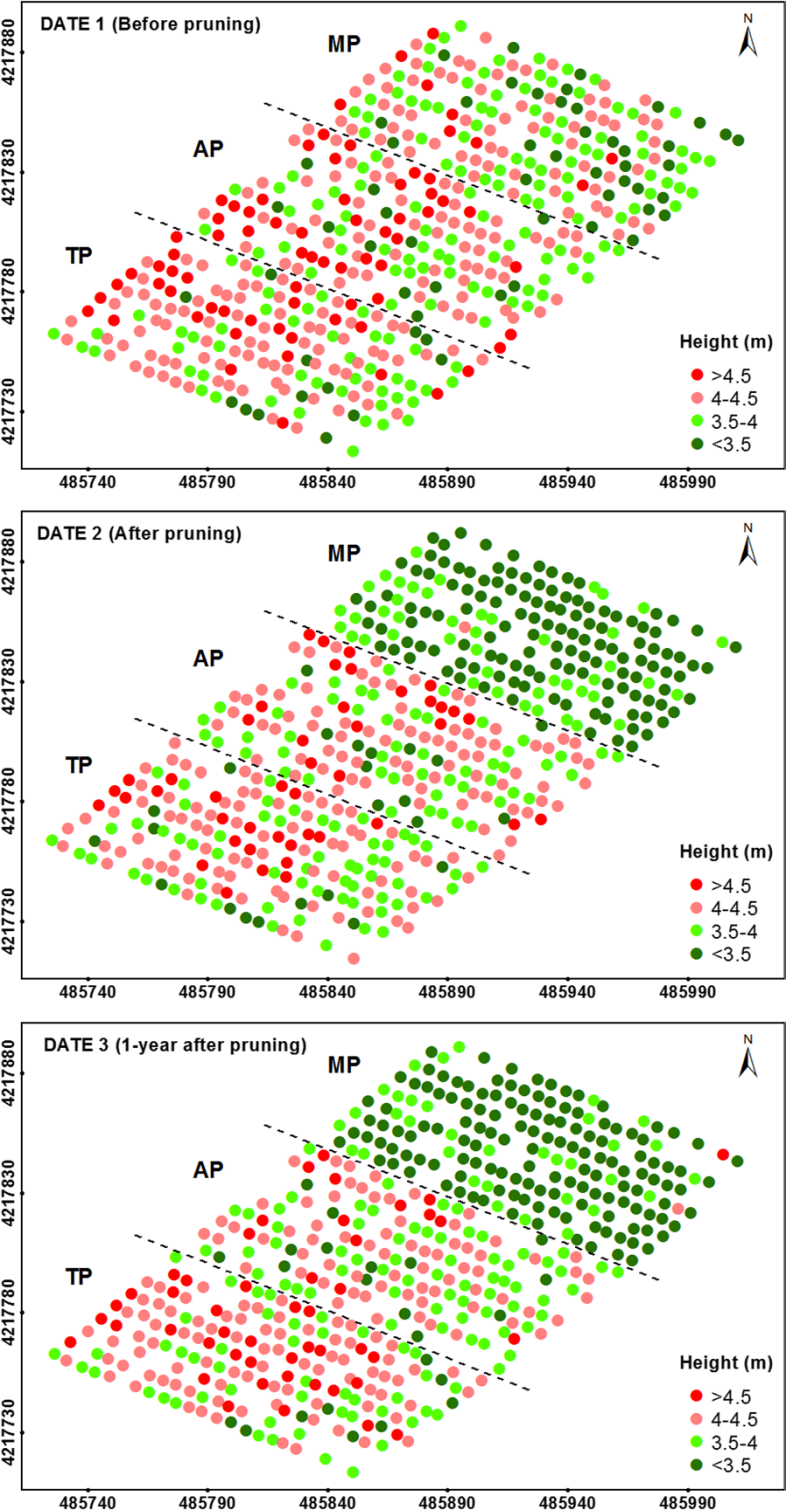
Four-level representation of the tree heights as computed on the three study dates. The *letters* indicate the pruning treatments as follows: TP (traditional), AP (adapted), and MP (mechanical). In the axes, coordinate system UTM zone 30 N, datum WGS84

**Fig. 4:**
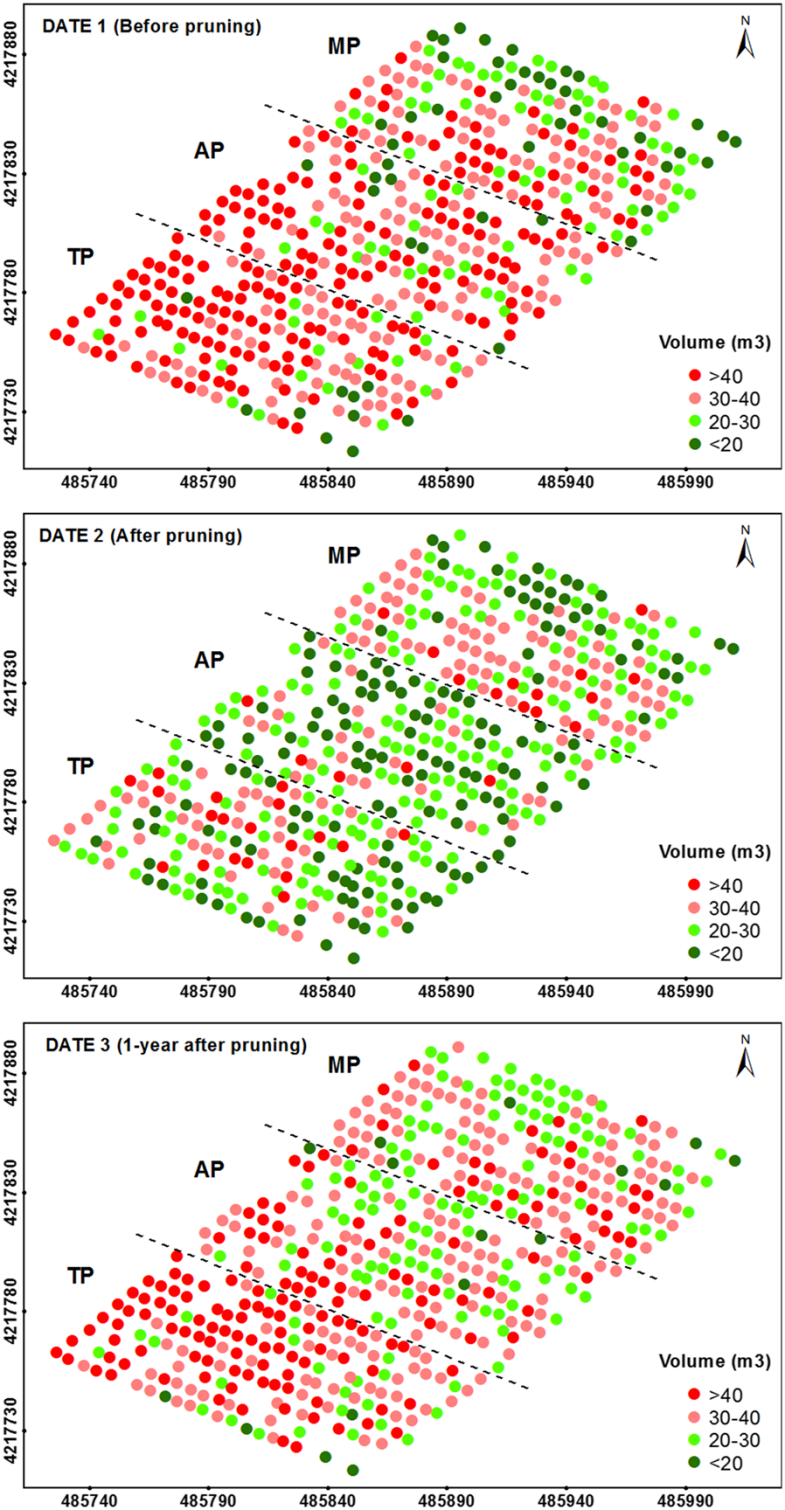
Four-level representation of the tree crown volumes as computed on the three study dates. The *letters* indicate the pruning treatments as follows: TP (traditional), AP (adapted), and MP (mechanical). In the axes, coordinate system UTM zone 30 N, datum WGS84

The detailed information reported in Table 1 was ranked as four levels of projected canopy area (Fig. 2), tree height (Fig. 3) and crown volume (Fig. 4), which allowed for the observation of all the tree variability at the orchard scale.

A view of these figures on date 1 (before pruning) revealed the distribution of the tree sizes throughout the orchard, showing a higher number of small trees located at the three upper rows of the graph, which corresponded to the top zone of the field. On date 2 (after pruning), the homogeneity of the tree heights was clearly notable at the MP parcel (Fig. 3), as shown by a coefficient of variation (CV) of only 6%. On this date, a severe reduction of the projected canopy areas and crown volumes was also observed on the trees located at the AP and TP zones. Next, the geometric features on date 3 (1-year after pruning) of a majority of the trees showed similar values to the one observed on date 1 for the projected areas and crown volumes (Figs. 2, 4, respectively) and on date 2 for the tree heights (Fig. 3). This finding indicates that the olive trees grew in area and volume over the duration of this experiment, but not in height.

### Agronomic objectives: Impact of every pruning treatment on the tree architecture, annual tree growth and tree restoration

**Fig. 5:**
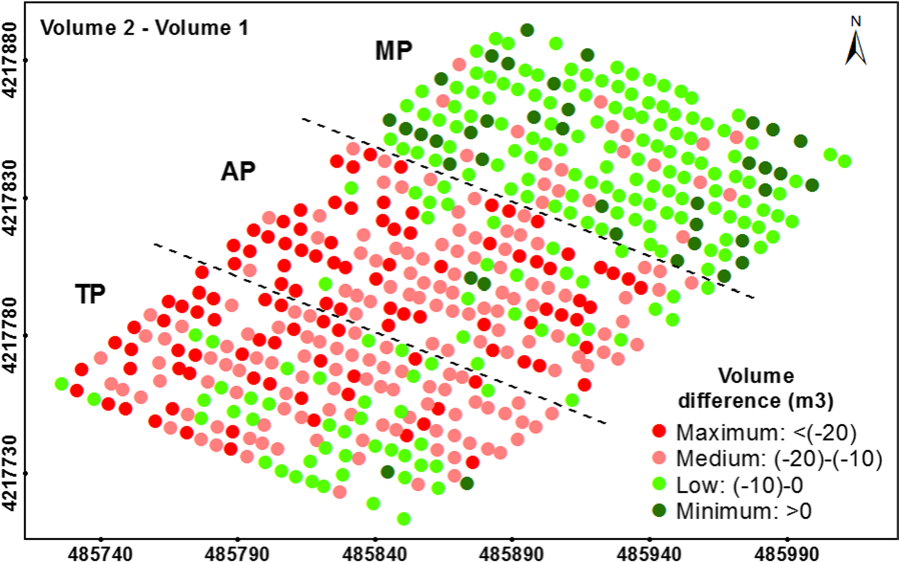
Four-levels representation of the pruning impact on tree volume (differences in tree volume between dates 2 and 1). The *letters* indicate the pruning treatments as follows: TP (traditional), AP (adapted), and MP (mechanical). In the axes, coordinate system UTM zone 30 N, datum WGS84

The impact of the pruning treatments on the tree architecture was evaluated by comparing tree dimensions on date 2 (after pruning) and date 1 (before pruning) (Fig. 5; Table 3). The AP treatment was generally the most aggressive for the olive trees, producing an average decrease of approximately 40% in canopy area and crown volume. However, the heights of the AP trees only decreased by an average of 0.05 m. These values were slightly greater than the ones obtained for the TP treatment. Regarding the MP treatment, the heights of these trees decreased 0.51 m on average, being up to ten times the overall decrease relative to the other two treatments, although the average reductions in the projected canopy area and crown volume were approximately five and four times lower than they were for the AP trees, respectively.

**Table 3:** Impact of tree pruning treatment on the tree architecture, when computed as the differences in the projected canopy area, tree height, and crown volume between dates 2 (after pruning) and 1 (before pruning)

Pruning treatments	Area (Date 2 − Date 1)		Height (Date 2 − Date 1)		Volume (Date 2 − Date 1)	
	Mean ± SD (m^2^)	%^a^	Mean ± SD (m)	%^a^	Mean ± SD (m^3^)	%^a^
Mechanical	−0.89 ± 1.40	−6.97	−0.51 ± 0.40	−12.15	−3.79 ± 4.93	−9.89
Adapted	−5.56 ± 2.68	−38.95	−0.05 ± 0.38	−0.65	−17.46 ± 8.73	−42.05
Traditional	−5.00 ± 2.87	−33.02	−0.03 ± 0.31	−0.37	−15.58 ± 8.19	−35.72

^a^Average percentage of increase (+) or decrease (−) of each tree geometric feature between the date 2 and the date 1, as follow: % = (feature_date2 − feature_date1)/(feature_date1)

**Fig. 6:**
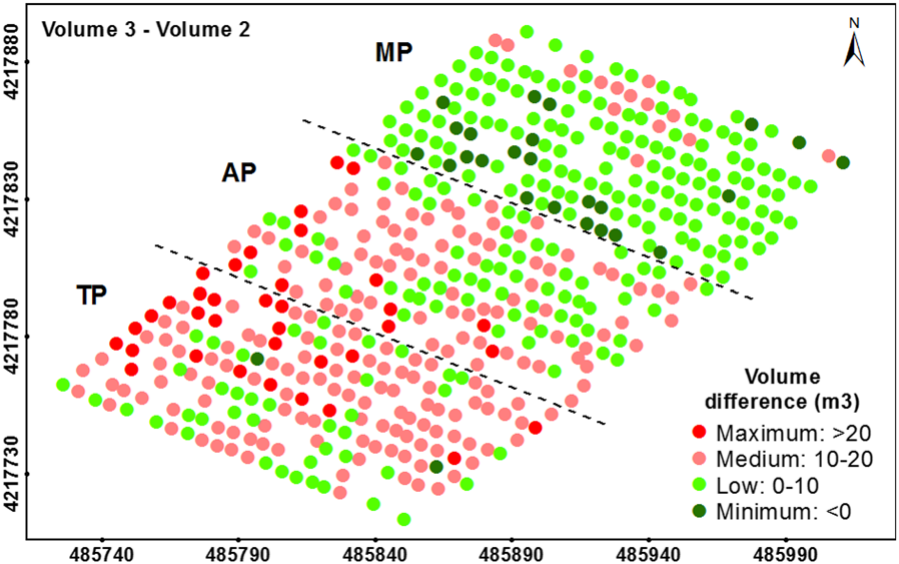
Four-level representation of the annual growth on tree volume after the pruning task (differences in tree volume between dates 3 and 2). The *letters* indicate the pruning treatments as follows: TP (traditional), AP (adapted), and MP (mechanical). In the axes, coordinate system UTM zone 30 N, datum WGS84

Regarding the impact of the pruning treatments on the annual tree growth (date 3–date 2), the type of pruning treatment might have a major influence on the vegetative response of the trees over time, mostly with respect to the crown volume growth (Fig. 6; Table 4). In comparing the tree data computed on date 2 and date 3 (after 1 year), the TP trees showed the greatest growth rates in projected canopy area (5.39 m^2^, 64.27%), tree height (0.06 cm, 1.66%), and crown volume (13.90 m^3^, 61.49%), although the projected canopy area of the AP trees showed higher growth in their percentage values (5.34 m^2^, or 71.14%). By contrast, the MP showed the lowest growth rates for the three variables (3.25 m^2^, −0.08 m and 4.66 m^3^, respectively).

**Table 4:** Impact of tree pruning treatment on annual tree growth, computed as the differences of projected canopy area, tree height, and crown volume between the date 3 (1-year after pruning) and the date 2 (after pruning)

Pruning treatments	Area (Date 3 − Date 2)		Height (Date 3 − Date 2)		Volume (Date 3 − Date 2)	
	Mean ± SD (m^2^)	%^a^	Mean ± SD (m)	%^a^	Mean ± SD (m^3^)	%^a^
Mechanical	3.25 ± 1.50	37.37	−0.08 ± 0.19	−2.32	4.66 ± 4.60	23.65
Adapted	5.34 ± 1.71	71.14	−0.05 ± 0.24	−1.14	11.58 ± 4.98	58.51
Traditional	5.39 ± 2.03	64.27	0.06 ± 0.20	1.66	13.90 ± 6.06	61.49

^a^Average percentage of increase (+) or decrease (−) of each tree geometric feature between the date 3 and the date 2, as follow: % = (feature_date3 − feature_date2)/(feature_date2)

**Fig. 7:**
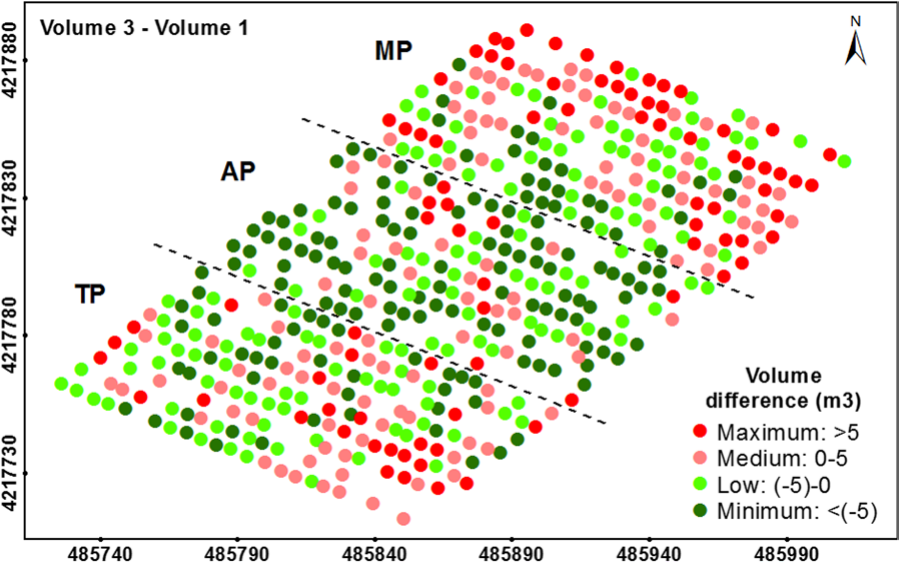
Four-level representation of the tree restoration in terms of volume (differences in tree volume between dates 3 and 1). The *letters* indicate the pruning treatments as follows: TP (traditional), AP (adapted), and MP (mechanical). In the axes, coordinate system UTM zone 30 N, datum WGS84

These results are linked to the ability of the trees to return to their initial dimensions from before the pruning task (Fig. 7; Table 5). Of the three treatments applied here, most MP trees were totally restored in terms of canopy area and volume in comparison to the original tree dimensions before the pruning task (date 1), but not in terms of the tree height. On average, the trees exceeded the canopy area and crown volume by 2.35 m^2^ and by 0.87 m^3^, respectively, although these trees were 0.59 m smaller in comparison to their heights on date 1. In the case of TP, these trees generally grew back to their original dimensions regarding the canopy area (0.39 m^2^ of average excess) and height (0.03 m of average excess), but not in terms of crown volume (1.68 m^3^ of average shortage). Finally, the AP trees did not generally reach their initial canopy area, tree height or crown volume in most of the trees.

**Table 5:** Impact of the tree pruning treatment on tree restoration, when computed as the differences in the canopy area, tree height, and crown volume between date 3 (1-year after pruning) and date 1 (before pruning)

Pruning treatments	Area (Date 3 − Date 1)		Height (Date 3 − Date 1)		Volume (Date 3 − Date 1)	
	Mean ± SD (m^2^)	%^a^	Mean ± SD (m)	%^a^	Mean ± SD (m^3^)	%^a^
Mechanical	2.35 ± 1.76	26.14	−0.59 ± 0.42	−14.26	0.87 ± 6.47	10.26
Adapted	−0.23 ± 2.50	1.95	−0.10 ± 0.36	−1.99	−5.88 ± 7.81	−10.76
Traditional	0.39 ± 2.32	6.68	0.03 ± 0.28	1.01	−1.68 ± 6.80	0.29

^a^Average percentage of increase (+) or decrease (−) of each tree geometric feature between the date 3 and the date 1, as follow: % = (feature_date3 − feature_date1)/(feature_date1)

**Fig. 8:**
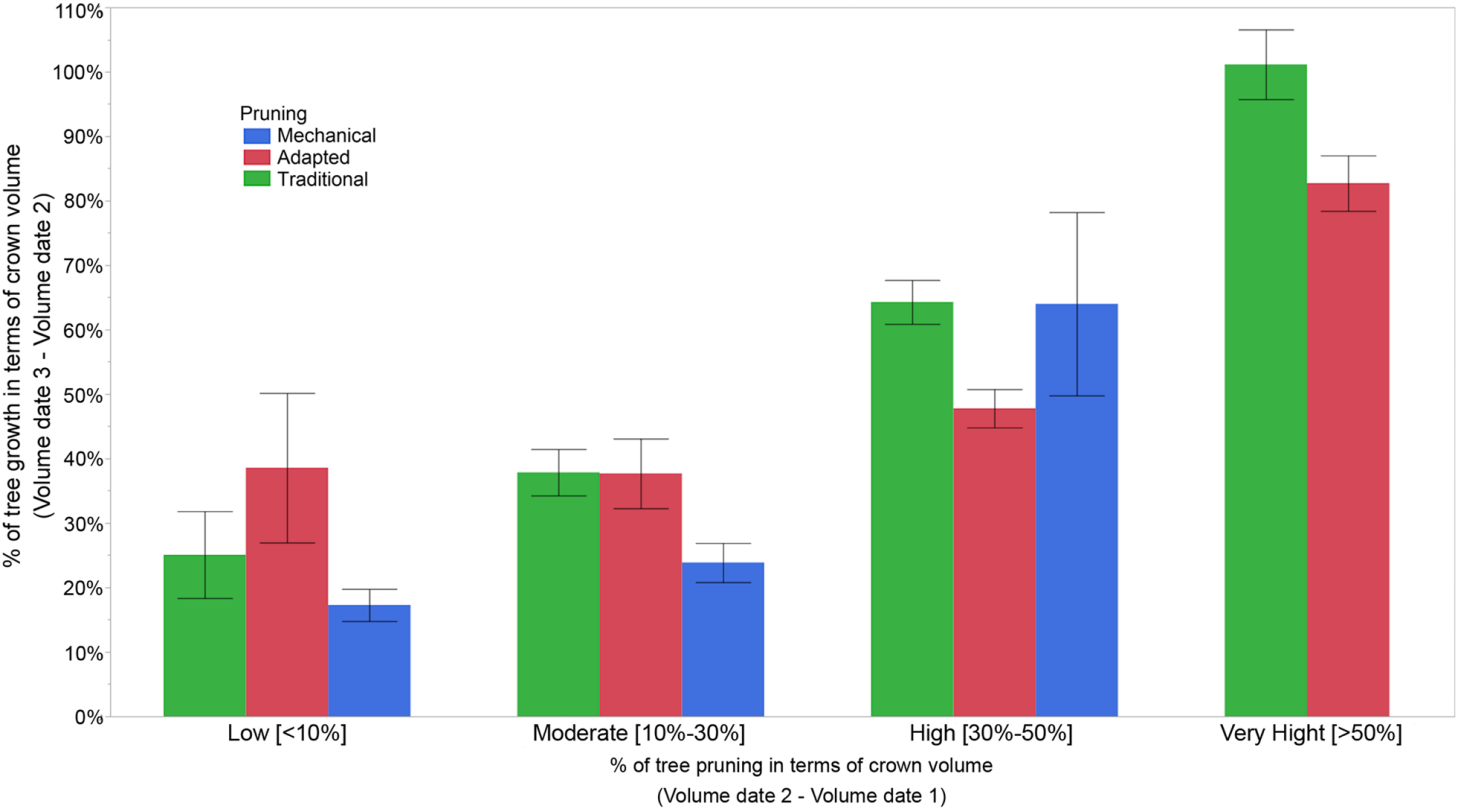
Percentage of annual tree growth after pruning as affected by the pruning severity and type of pruning treatment. *Lines* over the *columns* indicate the standard deviations

A detailed analysis of the data by grouping the trees according to pruning intensity revealed that tree restoration might be affected not only by the pruning severity but also by the type of pruning treatment (Fig. 8). In general, the trees that were subjected to more aggressive pruning experienced much more vegetative development for the three studied treatments. Moreover, it was determined that the trees that were pruned to less than 30% of their crown volume grew approximately 20–40% over one year, while the trees that were pruned by up to 50% of their crown volume grew more than 75% for the same period. However, differences in tree growth were also observed among pruning treatments. Our results showed that the AP trees were relatively more productive in terms of vegetative growth than the other two treatments when the pruning intensity was low (<10%) and similar to that of the TP trees when the intensity was moderate (10–30%). By contrast, the TP generated more vegetative growth when the pruning intensity was very high (>50%), and similar to the MP when the intensity was high (30–50%).

## Discussion

The direct assessment of the primary dimensions and certain geometric features of the olive trees such as the projected canopy area, height and crown volume is currently possible with the combined use of UAV imagery and advanced image processing and analysis procedures. This technology opens new opportunities to monitor tree status and progress at the field scale, as an efficient, objective and accurate alternative to the arduous and frequently inconsistent manual measurements on the ground [5]. This investigation takes advantage of this innovative UAV-based technology to evaluate the impact of pruning treatments in an olive plantation, reporting quantitative data for an unprecedented number of trees.

From the multi-temporal analysis of the location and the 3D models of every tree in the olive orchard, the effect of the pruning intensity on the tree architecture and annual tree growth after pruning was quantified. AP and TP reduced the crown volume by approximately 42 and 36% on average, respectively, which is four and fivefold more compared to that of MP (approximately 10% on average). However, the trees that were subjected to TP grew slightly more than the AP trees, accounting for crown volume increases of approximately 62 and 59% on average, respectively. Regarding the effect of pruning on tree heights, important differences were only reported in the MP trees, which were homogenously cut to 3.5–4 m over the surface, as observed in Fig. 3. The data observed in this figure also confirmed the capacity of this UAV-based technology to accurately measure the tree heights. The trees also experienced minimum changes in terms of vertical growth after the pruning treatments, suggesting that the tree primarily grew along the horizontal axes. As expected, the trees that were subjected to more severe pruning generally experienced higher growth over time, although the magnitude of this event was affected by the given pruning treatment. In comparing the three pruning treatments, it was observed that AP benefitted tree development if the pruning intensity was lower than 10% of the crown volume, although this treatment was relatively worse in terms of tree growth when the pruning intensity was higher than 30%. These overall results were obtained in adult irrigated trees, so further analysis are needed to support such agronomic conclusions in other scenarios, e.g., under rain-fed conditions or with younger plantations.

Together with the evaluation of the pruning treatments, remote sensed information about the tree architecture at the orchard scale also has multiple implications for tree physiology, agronomy and field management [25] with potential applications for investigations about tree growth and yield [4], crown porosity [26], or the interception of solar irradiation [27, 28], among others. For example, the results presented here would improve the prediction models that connect the tree crown volume and tree canopy density with the tree yields, which is a complex issue since these models depend on a large number of factors [29]. Some investigations have also addressed the relationship of pruning treatments to tree productivity and mechanical harvesting [30]. Tombesi et al. [31] studied the influence of the canopy density on the efficiency of a trunk shaker after applying several pruning intensities, and they concluded that moderate and heavy annual pruning assisted mechanical harvesting. By contrast, a lack of pruning caused the crown to grow upward and away from the primary branches which resulted in defoliation due to a lack of light and from parasitic attack.

Additionally, quantifying the impact of pruning on the tree volume gives an estimated value of the available residual biomass [32], which could serve to calculate the potential energy from this raw material [33] or, furthermore, to evaluate the site-specific effects of the application of these by-products on the soil in no-till systems to prevent land degradation and improve the organic matter content [34–36]. The use of pruned residues as mulch is growing [37] and can help prevent pollutant dispersion in olive groves [38].

Geo-referenced maps with the locations and dimensions of every tree could also be the basis for designing a programme for a variable rate application of plant protection products [39], and in combination with on-ground equipment [40, 41], contribute to help fulfill the requirements of the European Directive for a Sustainable Use of Pesticides [42].

## Conclusions

This investigation combined aerial images that were collected with an UAV on three different dates (before pruning, after pruning and 1-year after pruning), 3D models of the olive tree field that were created by photo-reconstruction procedures and an original OBIA algorithm to evaluate the impacts of three different pruning treatments (traditional, adapted and mechanical) on hundreds of irrigated trees. The projected canopy area, tree height and crown volume were quantified and compared among pruning treatments and flight dates.

The full procedure had high accuracy, and it correctly identified and measured every olive tree on the three dates with the exception of some cases when the 3D point cloud was incorrectly generated. As a general trend, the trees that were subjected to AP showed the highest foliage losses after pruning, followed by trees under TP. However, trees under TP experienced higher growths than the other trees for the quantification of this vegetative response one year after pruning. Due to the typical MP typology, the trees under this treatment maintained a more constant vegetative growth during this study.

This research offers valuable information for designing site-specific olive tree management strategies in the context of precision agriculture, which allows for the optimized application of agronomic tasks such as pruning, fertilization, pesticide use or irrigation, with the consequent economic and environmental benefits. The technology presented here can be made adaptable and transferable with corresponding adjustments to other woody crops such as vineyards or fruit orchards.

## Methods

### Study area and description of the pruning treatments

This research was performed in a commercial 20-year-old olive grove located in Villacarrillo, in the province of Jaen (southern Spain, central coordinates 485,885 m X, 4,217,810 m Y, system UTM zone 30 N, datum WGS84). A rectangular field of approximately 3 ha that was under drip-irrigation was selected for the experiment. This field was made up of 648 olive trees of the Arbequina variety, and it was laid out as 27 trees long and 24 across the field, with an intensive single-tree pattern of 8 × 4 m tree spacing. The field soil was loamy and silty clay loam, with at least 1.5 m deep without stoniness, and no limitations for crop production. The irrigation was controlled at a dose of approximately 800 m^3^ per hectare, which corresponded to 3200 l per tree. A natural cover crop, 1.5 m wide and composed of grass and legume species, covered the soil among the tree lines. The cover crop was controlled with a brush-cutter, meanwhile herbicides were applied in early autumn and spring to control weeds under the olive trees. Fertigation was applied at 100 units of fertilizer per hectare.

**Fig. 9:**
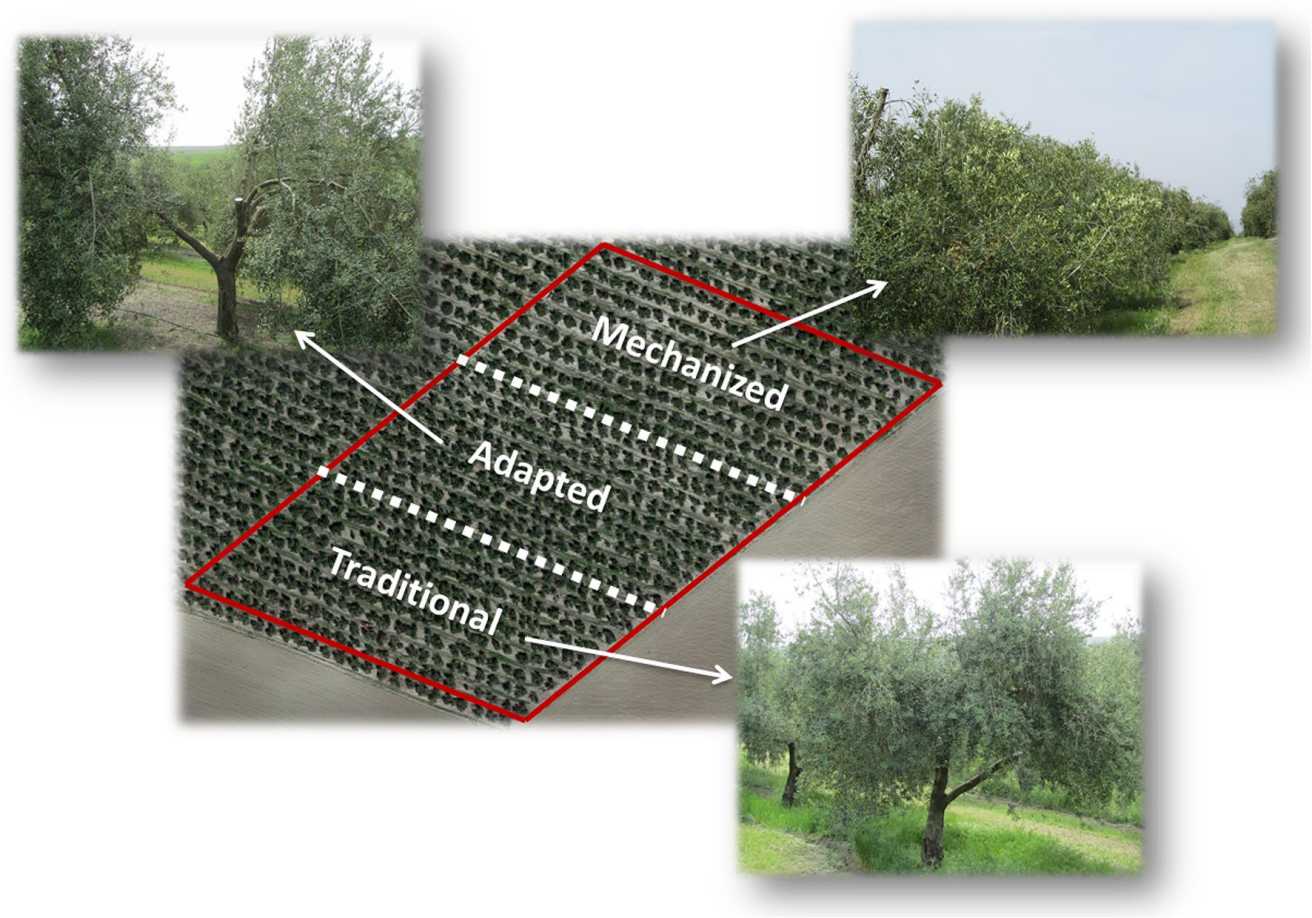
Description of the three pruning treatments evaluated in this investigation

The pruning strategy was part of a broader research program with the aim of studying the efficiency of different olive pruning and mechanical harvesting systems. Therefore, a simple demonstration strip design was selected in order to prioritize viability of mechanical pruning, which relies on continuous work at large areas, instead of designing a complex field experiment. The study field was divided in three sub-plots of 9 × 24 trees each, in which traditional, adapted and mechanical pruning treatments were separately performed on March 4th and 5th, 2015 (Fig. 9). In the TP, the highest branches, the crossed ones, and the ones below the base of the canopy and established at 60 cm over the soil were pruned. In the AP, the inner branches were totally removed, plus the crossed and low branches as described in the previous treatment. Thus, a large number of trees under this treatment presented a sizeable gap in the central crown part. The AP mainly aimed to adapt the olive architecture for canopy shaker harvesting. Finally, in the MP, a tractor with opposite mechanical cuts at a 30º angle removed the branches from 3.5 to 4 m above the terrain. This tractor also used a horizontal mechanical cut to remove the branches at less than 70 cm above the terrain.

### Multi-temporal UAV flights and the generation of geo-spatial products

**Fig. 10:**
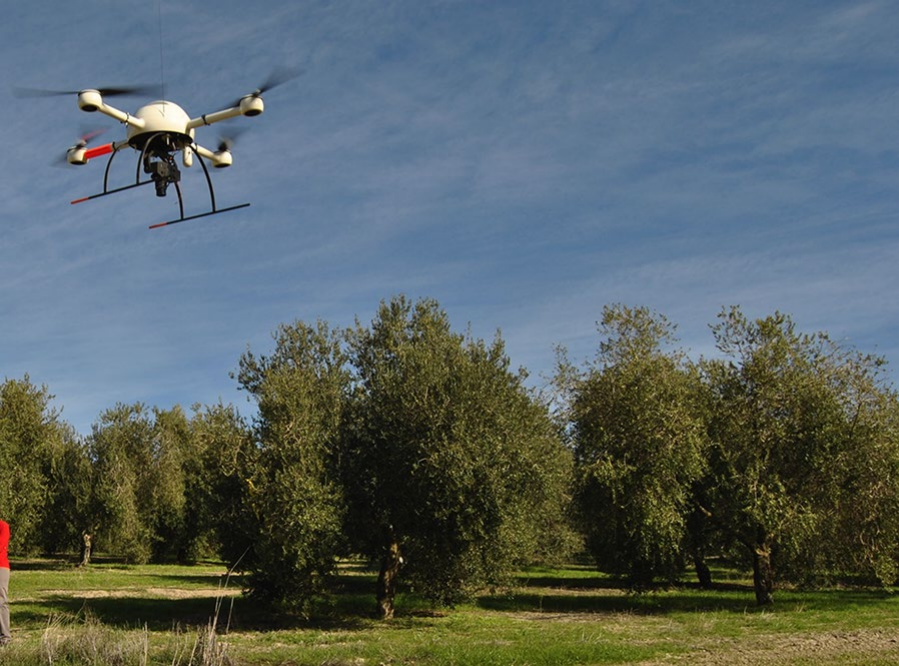
The quadcopter UAV and the Red–Green–Blue (RGB) camera used to acquire the remote images of the olive trees

A set of remote images of the experimental field were acquired through UAV flights that were performed on the following three dates: (1) before tree pruning (date 1: December 9th, 2014), (2) a short time after tree pruning (date 2: April 14th, 2015), and (3) almost a year after tree pruning (date 3: February 1st, 2016). The flight equipment was a quadcopter UAV with vertical take-off and landing model MD4-1000 (Microdrones GmbH, Siegen, Germany), with a still point-and-shoot camera of model Olympus PEN E-PM1 (Olympus Corporation, Tokyo, Japan) (Fig. 10). This camera took 12.2 megapixel images in true color (Red, R; Green, G; and Blue, B, bands) with 8-bit radiometric resolution and at a 14 mm focal length. The camera’s sensor size was 17.3 × 13.0 mm and the pixel size was 0.0043 mm. These parameters are needed to calculate the image resolution on the ground or, i.e., the ground sample distance as affected by the flight altitude.

During each flight, the UAV route was designed to take photos continuously at 1-s intervals, resulting in a forward lap of at least 90%. In addition, a side lap of 60% was programmed. The flight speed was 3 m/s and the flight altitudes were 100 m on the first and third dates and 50 meters on the second date. Due to the strong windy conditions, the 100-m flight was aborted on the second date. The UAV used a total of 24 and 15 min to fly the experiment field at 50 and 100 m altitude, respectively. The area covered in each flight was 3.5 ha. To fully cover the experimental field, the camera collected 840 and 420 RGB images at ground sample distances of 1.90 and 3.81 cm/pixel for 50- and 100-m flight altitudes, respectively. The flight operations fulfilled the list of requirements established by the Spanish National Agency of Aerial Security, including the pilot license, safety regulations and limited flight distance [43]. The UAV flights were authorized by the person in charge of the olive grove as well.

**Fig. 11:**
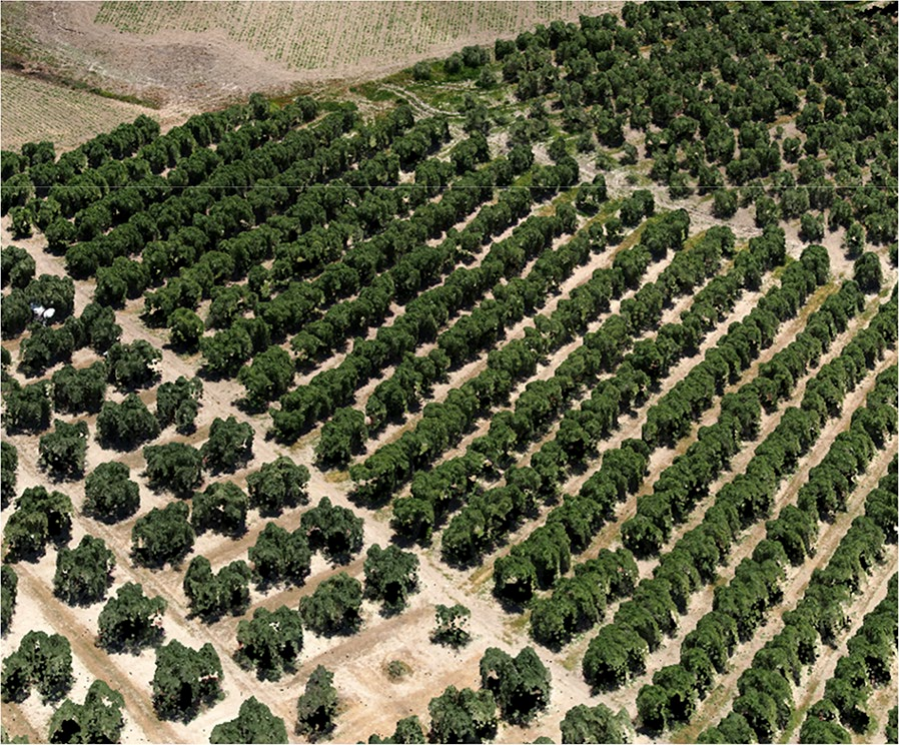
A partial view of the 3-D Point Cloud for the olive grove studied in this investigation, which was produced by the photogrammetric processing of the remote images taken with the UAV

In processing the set of UAV aerial images, the following three geo-spatial products of the olive grove were produced: (1) the 3D point cloud file, by applying the structure-from-motion technique (Fig. 11), (2) the DSM, with height information, which was created from the 3D point cloud, and (3) the ortho-mosaicked image, with RGB information on every pixel. In this research, Agisoft PhotoScan Professional software, version 1.2.4 build 2399 (Agisoft LLC, St. Petersburg, Russia) was used for this task. The mosaicking process was fully automatic with the exception of the manual localization of six ground control points that were used to georeference the products. These ground control points were located in the corners and the center of the olive orchard, and their coordinates were taken with a GPS device after the flight operations. The automatic process involved the following three phases: (1) aligning images, (2) building field geometry, and (3) ortho-photo generation. The common points and the camera position for each image were located and matched, which facilitated the refinement of the camera calibration parameters. Once the images were aligned, the point cloud was generated. Next, the DSM was built on the basis of the estimated camera position and the images themselves. This process requires high computational resources and it can usually take approximately 5–6 h due to the use of many high-resolution images. Finally, the images were projected over the DSM, and the ortho-mosaicked image was generated. The DSM is a 3D polygon mesh that represents the overflown area and reflects the irregular geometry of the ground and the tree crowns. The DSM was joined to the ortho-mosaic in the form of TIFF files, which produced a 4-band multi-layer file (Red, Green, Blue and DSM). More information about the PhotoScan function is described in Dandois and Ellis [44].

### Object-based image analysis algorithm for computing the 3D tree features

**Fig. 12:**
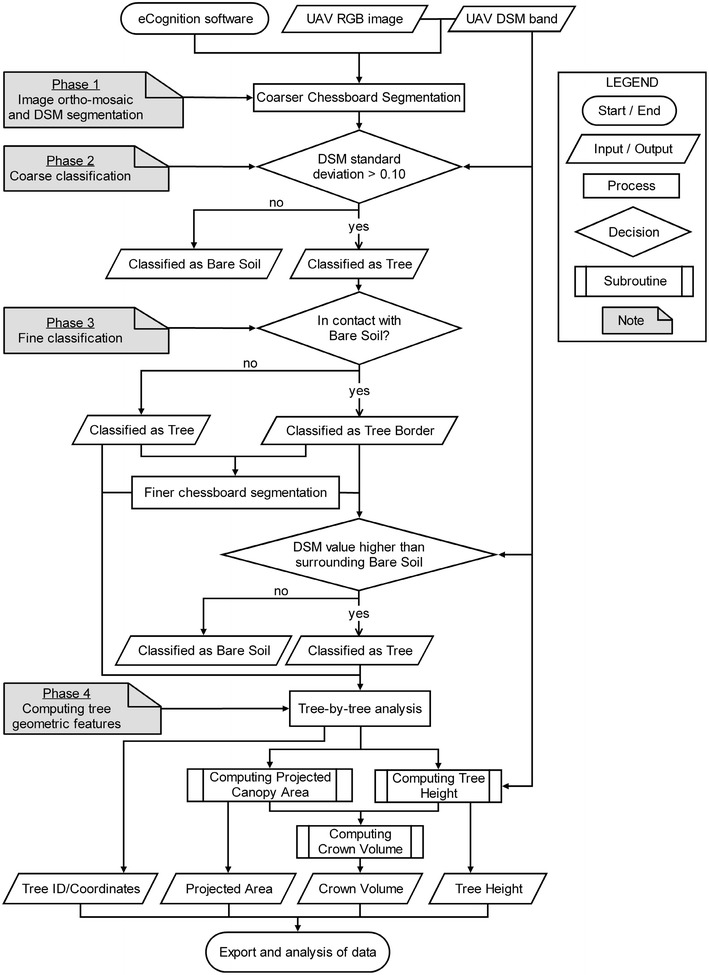
Flowchart of the OBIA algorithm developed in this investigation

An innovative algorithm based on the OBIA paradigm was applied to the 4-band multi-layer file that was created during the previous stage to classify and identify every individual tree in the olive grove and to compute the tree geographic position and primary 3D geometric features, including the projected canopy area, the tree height and the crown volume. The OBIA algorithm was developed using eCognition Developer 9 software (Trimble GeoSpatial, Munich, Germany) and it was adapted from the basic version described in Torres-Sánchez et al. [15]. However, the procedure presented here was original and included improvements and variations related to the specifications of this research (Fig. 12).

**Fig. 13:**
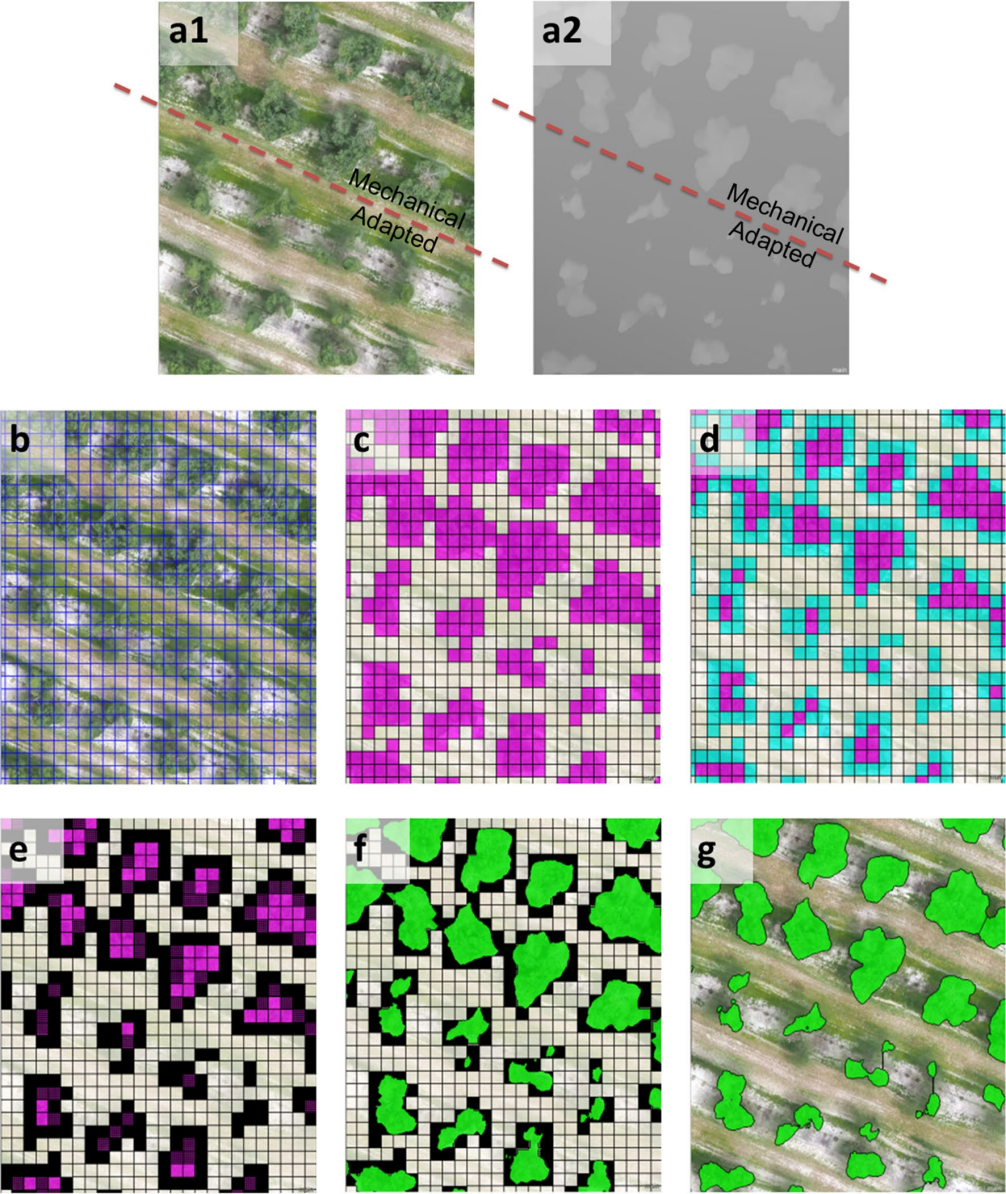
Partial views of the primary OBIA algorithm outputs: **a** the 4-band multi-layer file with the RGB (**a1**) and the DSM (**a2**) layers, showing the results of mechanical and adapted pruning as applied to the trees on the two *top* and *bottom rows*, respectively; **b** chessboard segmentation output; **c** the coarse classification of the tree (*pink colour*) and bare soil (*white colour*) objects based on the difference in DSM (height) values; **d** the coarse classification of the tree borders (*blue color*); **e** pixel-based segmentation of the tree borders; **f** the fine classification of the tree (*green colour*) objects; and **g** the tree and the bare-soil objects were joined with separately. As a result of the whole procedure, the algorithm computed the 3D tree geometric features (projected area, height and volume) and exported the values as vector and table files for further analysis

The algorithm was specifically programmed to run in a fully automatic manner without the need for user intervention, and with the ability to be auto-adaptive to any olive grove, independent of the plantation pattern and of the given pruning treatment. The full procedure was composed of a sequence of phases (Fig. 13), which is described as follows:

*Phase 1, image ortho*-*mosaic and DSM segmentation* The 4-band (B, G, R, and DSM) multi-layer file (Fig. 13a1–a2) was segmented into 1-m^2^ square objects by using the chessboard segmentation process (Fig. 13b). Because segmentation is by far the slowest task of the full OBIA procedure, the algorithm used chessboard segmentation instead of the multi-resolution option. In addition, it was programmed to only use the DSM band as the reference for the segmentation, which weighted the variable “height” instead of the spectral information. This configuration produced a notable decrease in computational demand, and consequently, an increase in the processing speed, without penalizing the segmentation accuracy.

*Phase 2, coarse classification of trees and bare soil* The segmented objects, whose standard deviation (SD) value of the DSM (height) layer was greater than 0.10 m, were classified as trees. The remaining objects were classified as bare soil (Fig. 13c).

*Phase 3, fine classification of trees:* To refine the tree delineation, the tree objects were analyzed at the pixel level. Firstly, the tree objects that were in contact with bare soil were classified as tree border objects (Fig. 13d), and next, they were segmented at the pixel size (Fig. 13e). Then, the algorithm classified every tree border object as a tree or bare soil by comparing their DSM value to the surrounding bare soil and tree DSM values (Fig. 13f). Finally, the objects classified as trees were joined into single objects and identified as individual trees (Fig. 13g).

*Phase 4, computing the tree geometric features* The algorithm automatically calculated the geometric features (projected canopy area, tree height and crown volume) of all the tree objects by applying a looping process in which every tree was individually identified and analyzed. During this sequential process, the height of every tree was obtained by comparing its maximum DSM value to the average DSM values of a bare soil area with a 1 m buffer surrounding each tree. Simultaneously, the crown volume was calculated by adding up the volumes (by multiplying the pixel areas and heights) of all the pixels corresponding to every tree. Finally, the OBIA algorithm automatically exported the identification, location and the three primary geometric features of every tree as vector (e.g., shapefile format) and table (e.g., Excel or ASCII format) files for further analysis.

### Data analysis

The outputs delivered by the OBIA algorithm for the three study dates were subjected to descriptive analysis with JMP version 10 software (SAS Institute Inc., Cary, NC, USA). The impacts of pruning on the tree architecture and annual tree growth was separately evaluated at each pruning zone by analyzing tree-by-tree variability over time, i.e., by quantifying the differences in the three primary dimensions (projected area, tree height and crown volume) between dates 1 and 2 (the impact on the tree architecture) and between dates 2 and 3 (the impact on the annual tree growth). In addition, the ability of the trees to return to their original dimensions before the pruning task was quantified by comparing the data on dates 1 and 3. The field experimental design, which prioritized mechanical pruning viability rather than testing statistical hypothesis, allowed for ranking pruning treatments according to averaged values obtained in every study date and to trends observed over time.

## Abbreviations

3D: three dimensions
AP: adapted pruning
DSM: digital surface model
GPS: global position system
LiDAR: light detection and ranging
MP: mechanical pruning
OBIA: object-based image analysis
RGB: red-green–blue bands
SD: standard deviation
TP: traditional pruning
UAV: unmanned aerial vehicles
